# Efficient Transfection of Large Plasmids Encoding HIV-1 into Human Cells—A High Potential Transfection System Based on a Peptide Mimicking Cationic Lipid

**DOI:** 10.3390/pharmaceutics12090805

**Published:** 2020-08-25

**Authors:** Christopher Janich, Daniel Ivanusic, Julia Giselbrecht, Elena Janich, Shashank Reddy Pinnapireddy, Gerd Hause, Udo Bakowsky, Andreas Langner, Christian Wölk

**Affiliations:** 1 Institute of Pharmacy, Martin Luther University Halle-Wittenberg, Wolfgang-Langenbeck-Strasse 4, 06120 Halle (Saale), Germany; julia.giselbrecht@pharmazie.uni-halle.de (J.G.); or christian.woelk@medizin.uni-leipzig.de (C.W.); 2 Department of Pharmaceutics and Biopharmaceutics, University of Marburg, Robert-Koch-Str. 4, 35037 Marburg, Germany; shashank.pinnapireddy@pharmazie.uni-marburg.de (S.R.P.); UBakowsky@aol.com (U.B.); 3 Robert Koch Institute, Division 18: HIV and Other Retroviruses, Nordufer 20, 13353 Berlin, Germany; ivanusicd@rki.de (D.I.); elena.janich@gmx.de (E.J.); 4 Biocenter, MLU Halle-Wittenberg, Weinbergweg 22, 06120 Halle (Saale), Germany; gerd.hause@biozentrum.uni-halle.de; 5 Institute of Pharmacy, Pharmaceutical Technology, Faculty of Medicine, Leipzig University, Eilenburger Strasse 15a, 04317 Leipzig, Germany

**Keywords:** gene therapy, cationic lipids, large plasmids, transfection, membrane fusion, HIV

## Abstract

One major disadvantage of nucleic acid delivery systems is the low transfection or transduction efficiency of large-sized plasmids into cells. In this communication, we demonstrate the efficient transfection of a 15.5 kb green fluorescent protein (GFP)-fused HIV-1 molecular clone with a nucleic acid delivery system prepared from the highly potent peptide-mimicking cationic lipid OH4 in a mixture with the phospholipid DOPE (co-lipid). For the transfection, liposomes were loaded using a large-sized plasmid (15.5 kb), which encodes a replication-competent HIV type 1 molecular clone that carries a Gag-internal green fluorescent protein (HIV-1 JR-FL Gag-iGFP). The particle size and charge of the generated nanocarriers with 15.5 kb were compared to those of a standardized 4.7 kb plasmid formulation. Stable, small-sized lipoplexes could be generated independently of the length of the used DNA. The transfer of fluorescently labeled pDNA-HIV1-Gag-iGFP in HEK293T cells was monitored using confocal laser scanning microscopy (cLSM). After efficient plasmid delivery, virus particles were detectable as budding structures on the plasma membrane. Moreover, we observed a randomized distribution of fluorescently labeled lipids over the plasma membrane. Obviously, a significant exchange of lipids between the drug delivery system and the cellular membranes occurs, which hints toward a fusion process. The mechanism of membrane fusion for the internalization of lipid-based drug delivery systems into cells is still a frequently discussed topic.

## 1. Introduction

Gene therapy is currently being applied in clinical practice and is no longer a science fiction treatment. The European Medicine Agency (EMA) and the US Food and Drug Administration (FDA) have authorized the first gene therapeutics (e.g., Strimvilis^®^, Kymriah^®^, Luxturna^®^) [1]. Due to the development of the CRISPR/Cas9 technology, new potential therapeutic strategies against a panoply of diseases have arisen [2,3]. However, there are both old and new hurdles to realize gene therapeutic strategies. A prerequisite for a successful gene therapy is the efficient transport of therapeutic nucleic acid material into target cells. To overcome various cellular barriers and to protect nucleic acids from enzymatic degradation, special delivery systems are required [4]. In fact, viral vectors are the leaders in gene therapeutic clinical trials, with more than 67.0% focusing on them [5]. These high numbers are mainly explained by their high efficiency. Nevertheless, these vectors have substantial drawbacks, such as immunogenic potential, oncogenic residual risk, expensive production costs, and a capsid-limited loading capacity [6,7,8,9,10]. In order to solve these problems, non-viral vectors have been developed in the last few decades, with cationic polymers or cationic lipids as the main representatives [11,12]. Theoretically, there is no limit to loading capacity. However it is very difficult to transport large-sized plasmids into cells [13]. For some strategies in gene therapy, it is important to develop efficient vectors which can transfer large DNA constructs. For instance, the co-delivery strategy with large plasmid coding for sgRNA and Cas-9-mRNA is one strategy for CRISPR-Cas-9-mediated therapies [14]. This short communication focuses on the problem of transfection with large nucleic acid constructs. 

**Figure 1:**
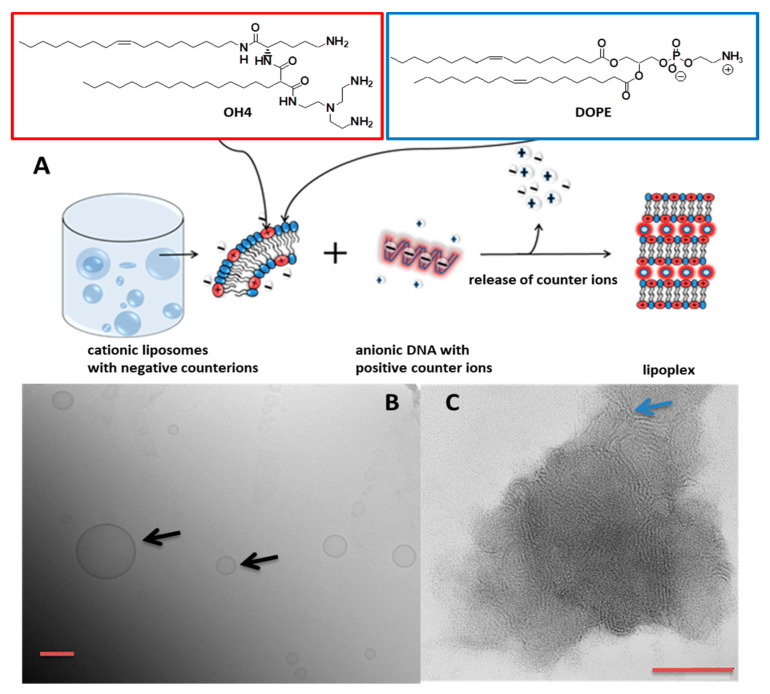
(**A**) Schematic illustration of the process of lipoplex formation. As lipids, the peptide-mimicking cationic lipid OH4 (*N*-{6-amino-1-[*N*-(9*Z*)-octadec-9-enylamino]-1-oxohexan-(2*S*)-2-yl-*N′*-{2-[*N*,*N*-bis(2-aminoethyl)amino]ethyl}-2-hexadecylpropandiamide) and the neutral co-lipid DOPE (1,2-dioleoyl-*sn*-glycero-3-phosphoethanolamine) were used. The scheme describes the complex formation between cationic lipid formulation and DNA. Both structures attract counter-ions which are released after complex formation, resulting in an increase in entropy. This gain of entropy and the electrostatic forces are the driving forces for the lipoplex formation. (**B**) Cryo-TEM micrograph of OH4/DOPE 1/1 (n/n) vesicles. The black arrows indicate liposomes. (**C**) Negative stain TEM of OH4/DOPE 1/1 (n/n) lipoplexes at N/P (ratio of the primary amino functions of the lipids to phosphate of the DNA) 4 complexing 4.7 kb pDNA. The blue arrow indicates the lamellar substructure of a lipoplex. The scale bars correspond to 100 nm.

The cationic portion of the lipid molecule interacts with negatively charged plasmid DNA, resulting in a liposome/nucleic acid complex (Figure 1), also known as a “lipoplex”, which has been described previously [15]. The drug delivery formulation presented in this report was prepared using the cationic lipid OH4, a representative of a class of highly potent peptide-mimicking cationic lipids, and the phospholipid DOPE (a co-lipid which improves efficiency due to the formation of non-lamellar mesophases [16,17]), employed in a molar ratio of 1:1 to yield cationic liposomes (Figure 1B) [18,19,20]. Subsequently, this liposome formulation was used to complex plasmid DNA (pDNA). Lipoplexes were further used to deliver pDNA into cells in the presence of 10% fetal bovine serum (FBS). This lipid formulation is capable of effectively transferring small (4.7 kb, as described earlier [18,19]) as well as large-sized plasmids (15.5 kb, as described in this report) into cells. The 15.5 kb pDNA codes for a replication-competent HIV type 1 molecular clone (HIV-1_JR-FL_Gag-iGFP) that contains a Gag-internal green fluorescent protein (iGFP), which is used in HIV research to investigate the mechanisms of viral assembly and cell–cell transmission of HIV particles [21,22,23,24]. For our purposes, the expressed Gag-iGFP is a reporter protein which is suitable for tracking distinct HIV budding sites at the plasma membrane.

## 2. Materials and Methods 

### 2.1. Materials

If not mentioned otherwise, chemicals were purchased from Sigma-Aldrich (Steinheim, Germany). DOPE and 1,2-dioleoyl-sn-glycero-3-phosphoethanolamine-N-(lissaminerhodamine B sulfonyl) (Rhod-DOPE) were bought from Avanti Polar Lipids (Alabaster, AL, USA). *N*-{6-amino-1-[*N*-(9*Z*)-octadec-9-enylamino]-1-oxohexan-(2*S*)-2-yl-*N′*-{2-[*N*,*N*-bis(2-aminoethyl)amino]ethyl}-2-hexadecylpropandiamide (OH4) was synthesized as described previously [13]. The plasmid HIV-1_JR-FL_Gag-iGFP was provided by Dr. Benjamin K. Chen (Mount Sinai Hospital, New York). Plasmid isolation kit (endofree Maxiprep) was purchased from QIAGEN (Hilden, Germany). Plasmid DNA was transformed in *Escherichia coli* Stbl2 (Invitrogen GmbH, Darmstadt, Germany), and plasmid DNA was isolated following the manufacturer’s instructions. The purity of pDNA was verified by the absorbance-quotient A260/A280 and using agarose gel electrophoresis. HEK293T cells were acquired from the German Collection of Microorganisms and Cell Cultures (DSMZ, Braunschweig, Germany). Cell culture media and fetal bovine serum (FBS) were supplied by Biochrom (Berlin, Germany). PoPo^®^-1 Iodide (1 mM in DMSO), was obtained from Thermo Fischer Scientific (Henningsdorf, Germany). The non-coding 15.5 kb plasmid DNA was purchased from Sigma-Aldrich.

### 2.2. Methods

#### 2.2.1. Preparation of Cationic Vesicles

Vesicles were prepared using the film hydration procedure. OH4 and DOPE were separately dissolved in chloroform/methanol (8/2, *v*/*v*) and mixed to a molar ratio of 1/1. The solvent was evaporated for 1 h at 200 mbar and a further 3 h at 10 mbar using a temperature of 50 °C. After the formation of dry lipid films, a sterile filtrated 10 mM MES buffer solution (pH 6.5) was added to give a final concentration of 1 mg·mL^−1^. Afterwards, the lipid dispersions were incubated at 50 °C while shaking (1400 rpm) for 30 min (Eppendorf Thermomixer 5436, Eppendorf, Hamburg, Germany), followed by sonication at 37 kHz for 3 min at 25 °C.

#### 2.2.2. Lipoplex Preparation

Lipoplex mixtures were prepared by combining DNA (the amount depends on the kind of experiment) with the liposome preparation in a mixing ratio that yields N/P 4 (N/P ratio = ratio of the primary amino functions of the lipids to phosphate of the DNA). The plasmid DNA was added to the lipid dispersion in one step. The samples were incubated for 15 min at 25 °C while shaking at 1000 rpm.

#### 2.2.3. Transmission Electron Microscopy with Negative Staining (Negative Stain TEM)

Samples for negative stain were diluted to a final concentration of 0.05 mg·mL^−1^ with 10 mM MES buffer (pH 6.5). Subsequently, 5 μL of the samples was spread on copper grids coated with a Formvar film. After 1 min of incubation, the excess liquid was absorbed by a filter paper. Thereafter, the samples were washed with H_2_O and stained with 1% aqueous uranyl acetate. The dried specimens were examined using an EM 900 transmission electron microscope (Carl Zeiss Microscopy GmbH, Oberkochen, Germany). Micrographs were acquired using an SSCCD SM-1k-120 camera (TRS, Moorenweis, Germany).

#### 2.2.4. Cryogenic Transmission Electron Microscopy (CryoTEM)

The liposomes were prepared with a concentration of 2 mg·mL^−1^. Vitrified specimens were prepared using a blotting procedure, performed in a chamber with a controlled temperature and humidity using an EM GP grid plunger (Leica, Wetzlar, Germany). The sample dispersion (6 μL) was placed onto an EM grid coated with a holey carbon film (Cflat, Protochips Inc., Raleigh, NC, USA). Excess solution was then removed by blotting with a filter paper to leave a thin film of the dispersion spanning the holes of the carbon film on the EM grid. Vitrification of the thin film was achieved through rapid plunging of the grid into liquid ethane held just above its freezing point. The vitrified specimen was kept below 108 K during storage, transferred to the microscope, and investigated. Specimens were examined with a Libra 120 Plus transmission electron microscope (Carl Zeiss Microscopy GmbH, Oberkochen, Germany) operating at 120 kV. The microscope was equipped with a Gatan 626 cryotransfer system. Images were acquired using a BM-2k-120 dual-speed on-axis SSCCD camera (TRS).

#### 2.2.5. Particle Size and Charge Measurements

Dynamic light scattering (DLS) experiments for the particle size determination were measured with a Zetasizer Nano ZS ZEN3600 (Malvern Instruments, Worcestershire, UK). The scattering angle was 173°. Three measurements consisting of 15 runs with a duration of 20 s for each run were performed at 25 or 37 °C. For the calculations, a viscosity of η = 0.8872 mPa·s and a refractive index of 1.33 were considered. Zetasizer Software 6.34 was used to fit the correlation function to calculate z average diameter and polydispersity index (PDI).

The zeta potential measurements were performed via laser Doppler velocimetry using a Zetasizer Nano ZS ZEN3600 (Malvern Instruments, Worcestershire, UK) and a clear disposable folded capillary cell (DTS1060, Malvern Instruments). Three independent measurements (30 runs) with a voltage of 60 V were performed at 25 °C. For the calculations, the viscosity (η = 0.8872 mPa·s), dielectric constant (ε = 78.5 F m^−1^) and refractive index (n = 1.33) of water were assumed. The mobility μ of the diffusing aggregates was converted into the ζ potential using the Smoluchowski relationship ζ = μ η/ε (Zetasizer Software version 6.34).

#### 2.2.6. Atomic Force Microscopy (AFM)

AFM was used to complement the particle size analysis performed by DLS. A total of 10 μL of the sample was pipetted onto silica wafers, which were glued onto glass slides. The samples were allowed to settle onto the wafers before being gently washed with water, followed by nitrogen gas. AFM was performed under ambient conditions using a Nanowizard^®^ 3 Nanoscience AFM (JPK Instruments, Berlin, Germany) in intermittent contact mode with an aluminum coated silicon nitride probe (HQ:NSC14/Al BS; Mikromasch, Tallinn, Estonia) at scan rates between 0.5 and 1 Hz, as described previously.

#### 2.2.7. Confocal Laser Scanning Microscopy (CLSM) Experiments

The fluorescence microscopic experiments were performed using a confocal laser scanning microscope (Zeiss LSM 780, Carl Zeiss, Oberkochen) using an inverse microscope (AxioObserver.Z1, Carl Zeiss, Oberkochen, Germany) with a fast z-scanning stage and 40 × and 63 × Plan APOCHROMAT objectives. Zeiss Software ZEN2011 was used for the generation of images, microscope control, and for data processing. We seeded 2 × 10^4^ HEK293T cells per 8-well ibidi^®^ µ-Slide cell culture well. At a confluence of 60%, cells were washed once with PBS buffer (pH 7.4) containing Ca^2+^ and Mg^2+^. Lipoplexes were added to the cells (0.2 μg plasmid DNA per well) and DMEM (Dulbecco’s Modified Eagle’s Medium) was added containing 10% FBS to a final volume of 200 μL per well. After 8 or 24 h, the cells were washed twice with PBS, followed by a fixation step using 0.2 mL 2% paraformaldehyde in PBS, and cells were incubated in the dark at room temperature for 15 min. The cells were then washed twice with PBS and leaved in 200 μL PBS. pDNA was tagged by the intercalating agent PoPo^®^-1 iodide (λ_ex_^max^ = 434 nm, λ_em_^max^ = 456 nm) in a base pair to dye ratio of 20. The labeled DNA was purified from non-intercalated dye using a MSB^®^ Spin PCRapace DNA purification kit (Stratec Molecular, Berlin, Germany). The virus budding sites were visualized by detecting florescence signals of eGFP (λ_ex_^max^ = 488 nm, λ_em_^max^ = 507 nm). Lipid labeled lipoplexes where prepared from liposomes consisting of OH4/DOPE/Rhod-DOPE (λ_ex_^max^ = 535 nm, λ_em_^max^ =560 nm) 1/1/0.002 (n/n/n). 

## 3. Results

**Figure 2:**
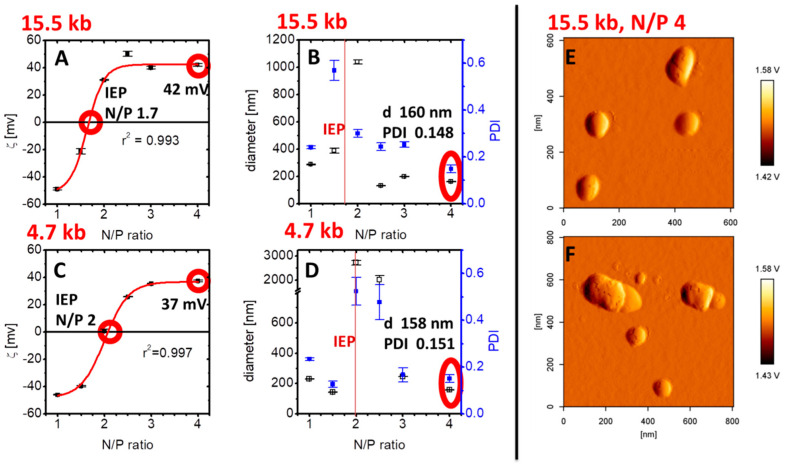
Zeta potential (mean and standard deviation of 3 measurements consisting of 30 runs, (**A**,**C**) and particle size (z- average diameter and the corresponding polydispersity index (PDI), mean and standard deviation of 3 measurements consisting of 15 runs, **B**,**D**) as a function of the N/P ratio, a value for the degree of loading the lipid formulation with DNA of different size: 15.5 kb (**A**,**B**) and 4.7 kb (**C**,**D**). Representative atomic force microscopy (AFM) images of OH4/DOPE 1/1 (n/n) in the complex with 15.5 kb DNA at N/P 4 are shown (**E**,**F**).

OH4/DOPE 1/1 (n/n) formed unilamellar liposomes without extrusion, confirmed by Cryo-TEM (Figure 1B). These liposomes complex nucleic acids due to electrostatic interactions, resulting in a reorganization of the lipid self-assembling and a formation of compact lipid/DNA complexes (lipoplexes) with a lamellar substructure (Figure 1C). Next, the influence of the size of the nucleic acid construct on the size and charge of the lipoplex was monitored. For this reason, we complexed a non-coding 15.5 kb DNA with the lipid formulation OH4/DOPE 1/1 (n/n) at different N/P ratios and compared the particle size and charge with lipoplexes loaded with the standard 4.7 kb DNA (Figure 2). The N/P ratio (ratio between the primary amines of the lipid to the phosphate groups of the DNA) indicates the degree of nucleic acid complexation. The charge of the lipoplexes as a function of the N/P ratio showed a sigmoidal shape for the lipoplexes composed of both DNA species (Figure 2A,C). Nevertheless, there were slight changes at characteristic points. First, the isoelectric point (IEP), indicating neutral charged lipoplexes (compensatory pairing of positive and negative charges), was at lower N/P ratios for the complex formation of OH4/DOPE with the large-sized DNA construct (N/P 1.7 for the 15.5 kb plasmid DNA and N/P 2 for the 4.7 kb plasmid DNA; compare Figure 2A,C). After the IEP, a positive plateau was reached. The lipoplex formulation of interest, OH4/DOPE 1/1 (n/n) N/P 4, was in this plateau and resulted in a zeta potential of 42 mV for the 15.5 kb DNA and 37 mV for the 4.7 kb DNA. Consequently, the zeta potential was 5 mV higher for the lipoplexes with the larger-sized DNA (Figure 2A,C) but both values were highly positive, and consequently, this small deviation should have no biological effects. The size of lipoplexes as a function of the N/P ratio is shown in Figure 2B,D. Near the IEP, particle size and PDI increased due to aggregation, but in the plateau region of the zeta potential curve, the diameter of the lipoplexes was below 400 nm. OH4/DOPE 1/1 (n/n) N/P 4 was characterized by lipoplexes with a diameter of 160 nm (PDI 0.148) for the 15.5 kb DNA and 158 nm (PDI 0.151) for the 4.7 kb DNA (compare Figure 2B,D). Consequently, the size of the lipoplexes was not affected by the size of the DNA at N/P 4. Additional AFM images serve as proof that the particle size is determined by DLS for the OH4/DOPE 1/1 (n/n) N/P 4 15.5 kb DNA (Figure 2E,F).

**Figure 3:**
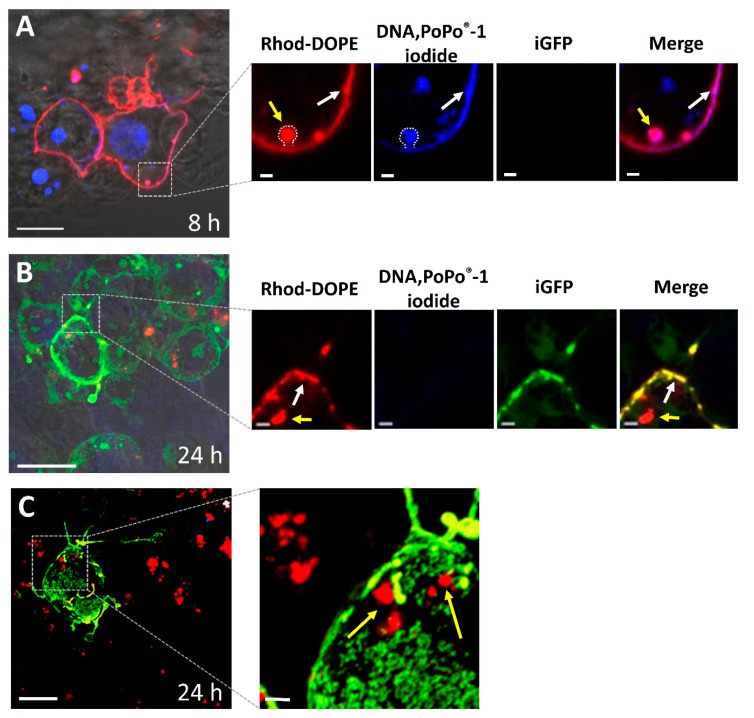
Confocal fluorescence microscopy images of HEK293T cells at 8 h (**A**) and 24 h (**B**,**C**) post-transfection using a lipid mixture containing OH4/DOPE 1/1 (n/n) in a complex with pDNA of the molecular clone HIV-1_JR-FL_Gag-iGFP at an N/P ratio of 4. To show typical three-color merged images, Rhod-DOPE (red), pDNA labelled with PoPo^^®^^-1 iodide (blue), and iGFP (green) are selected. Colocalization of Rhod-DOPE and pDNA-PoPo^^®^^-1 iodide in the cell membrane (white arrow) is displayed; Rhod-DOPE cell inclusions were marked with yellow arrows. Observed omega-like structures are marked with a dot line. After 24 h (**B**,**C**) post-transfection, the expression of the molecular clone HIV-1_JR-FL_Gag-iGFP is detectable (green), and viral budding formations can be observed on the surface (cut-out from C). The images were obtained with an inverted confocal laser microscope (Zeiss cLSM 780). For image (**C**), a z-stack of optical sections through the samples was visualized using Zeiss ZEN2011 software to generate a 3D projection. The scale bars represent a length of 10 µm, except in the image cut-outs, where they represent a length of 1 µm.

The efficient transfection of the 15.5 kb large-sized plasmid (molecular clone of HIV-1_JR-FL_Gag-iGFP) in the presence of 10% serum with the newly developed cationic lipid formulation was examined by detecting the eGFP fluorescence signals (Figure 3). Cell localization studies showed PoPo^®^-1 labeled pDNA within the cell nucleus. Furthermore, the lipid label was localized as a restricted domain inside the cell (red puncta) and in addition localized over the cell membrane of HEK293T cells (Figure 3). The observation of omega-like structures at 8 h post-transfection (Figure 3A, yellow arrow) is typical for lipoplex internalization by endocytosis [25,26]. Moreover, our observation indicates a membrane fusion between lipid formulation and cell membrane (lipid and DNA label at the cell membrane, Figure 3A, white arrow), a frequently discussed alternative mechanism of lipoplex uptake [27]. At 24 h post-transfection, the expression of the Gag-internal green fluorescent protein (Gag-iGFP) was detectable on the cell surface (Figure 3B, C). Interestingly, the labeled lipids were detected as restricted inclusion domains in the cytoplasm and are additionally accumulating in the cell membrane. 

## 4. Discussion

Generally, one obstacle in gene transfection is the low delivery efficiency of large-sized DNA constructs. In this context, Campeau et al. have shown a dramatic drop in transfection efficiency with plasmids of 12 kb or more [28]. Further, Kreiss et al. had similar results for the transfer of plasmids with a size between 2.9 and 52.5 kb into HeLa and NIH3T3 cells [13]. In addition, they reported that the lipoplex structure and size were not modified by increasing plasmid DNA length, a phenomenon that is also reported in this study. Lipoplex charge and size are comparable for OH4/DOPE in a complex with 4.7 and 15.5 kb DNA. A further critical factor in non-viral gene transfer is the presence of serum during the incubation of the nucleic acid delivery system with cells [29]. In this report, we show the efficient delivery of a 15.5 kb plasmid in the presence of 10% serum in HEK293T cells using the nucleic acid delivery system OH4/DOPE 1/1 (n/n). In recent reports, we were able to demonstrate the rapid uptake of complexes containing OH4/DOPE 1/1 (n/n) N/P 4 with a 4.7 kb plasmid in A549 cells, and endocytosis was identified as a main route of internalization of lipoplexes; nevertheless, evidence for membrane fusion processes was also found [30]. In this report, we also observed membrane fusion with lipoplexes (Figure 3A,B). The fluorescent staining of the cell membrane indicates lipid exchange processes between cationic lipid formulation and the cell membrane, which occurs during fusion processes. Whether the fusion processes occur directly at the cell membrane or in the endocytotic vesicles cannot be determined, but the late events during incubation have to be connected with a distribution of the fluorescent labeled lipid over the plasma membrane after vesicle trafficking back to the cell membrane, followed by vesicle–membrane fusion. Nevertheless, such evidence for membrane fusion was not present in all cells which internalized the DNA (Figure 3A) or expressed the HIV-1_JR-FL_Gag-iGFP construct (Figure 3B). Membrane fusion is not the only process of lipoplex internalization. The contribution of endocytic routes to the internalization of OH4/DOPE lipoplexes containing 15.5 kb DNA into HEK293T cells has not yet been investigated.

## 5. Conclusions

The focus of our work is a continuous development of nanostructured carrier systems composed of a new class of peptide-mimicking transfection lipids for potential medical use. In this report, we were able to demonstrate the ability of cationic lipid formulation OH4/DOPE 1/1 (n/n) to transfer a large-sized plasmid (15.5 kb) into HEK293T cells in the presence of serum. Moreover, independently of the length of the DNA (4.7 or 15.5 kb), small-sized lipoplexes with a diameter of around 160 nm (158 nm for 4.7 kb DNA vs. 160 nm for the 15.5 kb DNA) and a PDI around 0.150 (0.151 for the 4.7 kb DNA vs. 0.148 for the 15.5 kb DNA) could be developed. Successful transfection, resulting in the expression of viral proteins, was shown through the efficient budding of HIV. The presented lipid formulation was able to deliver several genes by transferring large-sized DNA constructs. A potential application of this delivery system could be in nucleic-acid-based vaccination.
